# Tuberculosis screening practices and outcomes in an australian dialysis unit

**DOI:** 10.1186/s12882-023-03304-0

**Published:** 2023-08-23

**Authors:** Hannah Wallace, Craig Nelson, Sandra Crikis

**Affiliations:** 1 Department of Nephrology, Western Health, 176 Furlong Road St Albans, St Albans, VIC 3021 Australia; 2 Department of Medicine, Western Health, University of Melbourne, Melbourne, VIC 3021 Australia; 3 Western Health Chronic Disease Alliance, Western Health, St Albans, VIC 3021 Australia; 4 Deakin University, Victoria, Australia

**Keywords:** Latent tuberculosis infection, Dialysis, Active tuberculosis, Quantiferon Gold screening

## Abstract

**Background:**

The World Health Organisation (WHO) recommends all dialysis patients undertake routine screening for latent tuberculosis infection (LTBI) in high income countries such as Australia. However, we employ a targeted screening approach in our Australian dialysis unit in line with local and some international guidelines. We analysed our practices to assess the validity of our approach.

**Methods:**

A retrospective review of new dialysis patients during the period 2012–2018 was undertaken. Patient records were reviewed for basic demographic data, comorbidities, LTBI screening using Quantiferon Gold (QFG), and outcomes, including episodes of active TB, to June 2020.

**Results:**

472 patients were included. WHO high risk country of origin patients accounted for 22% (n = 103). 229 patients (48.5%) were screened using QFG. The single main indication for screening was transplantation waitlisting. 34 patients had a positive QFG result. Active tuberculosis developed in two patients during the observation period. Both occurred in the screened cohort, the cases having previously tested negative via QFG at 11 and 16 months, prior to the development of active tuberculosis. No patients in the unscreened cohort developed active tuberculosis during the observation period. WHO high risk country of origin was associated with positive QFG status, odds ratio 10.4 (95% CI 3.3–31.2).

**Conclusion:**

The data failed to show a benefit from widening of the screening program within our dialysis unit. However, a much larger sample size will be required to confidently assess the impact of the current approach on patient outcomes. Analysis of current screening practices and outcomes across all Australian dialysis services is warranted to assess the risks and benefits of widening the screening practices to include all dialysis patients as recommended by the WHO.

## Background

The communicable disease tuberculosis (TB) is the second leading cause of death per year from a single infectious agent worldwide, only recently overtaken by Sars-CoV-2 [1]. Dialysis patients have an increased incidence of latent tuberculosis infection (LTBI) and progression to active TB with a 3-to-25-fold increase in risk [2–4]. The increase in risk is thought to be due to changes in the immune system in addition to co-morbidities and socio-economic factors [3–6].

Due to the increased risk, the World Health Organisation (WHO) recommends systematic testing and treatment for LTBI in dialysis patients of high to middle income countries [7]. In contrast other guidelines such as the British Thoracic Guidelines recommend a more targeted approach, utilising clinical history, examination and chest X-ray, with routine use of the tuberculin skin test (TST) or interferon gamma release assay (IGRA) not recommended for the indication of end stage renal failure alone [8–10].

Australia is a high-income country with low rates of endemic TB. Whilst the national position statement regarding the management of LTBI does not make specific recommendations with regards to dialysis patients, it does recommend identifying patients or populations at risk when considering implementation [10]. They suggest migrants from high incidence of TB country origin over the age of 35 with one or more risk factors (including end stage renal failure) be considered for testing, with prioritisation of recent migrants [11]. State guidelines vary, all recommended testing in those with both a high pre-test probability (such as individuals coming from endemic areas) and high risk of progression to active disease [12–15], with some but not all guidelines listing end stage renal failure and dialysis as a high-risk condition [13–15]. Notably both National and State guidelines all outline the importance of testing with intention to treat [10–15]. A previous Australian cohort registry study confirmed an increased risk for active TB in the dialysis population compared to the general population. Adjusting for age, TB incidence in country of origin, indigenous status and gender, the relative risk for TB in dialysis patients was 7.8 (95% CI 3.3–18.7), [4]. This raises the question of whether a targeted approach considering risk factors is sufficient, or a generalised screening program as proposed by the WHO, is more appropriate in the Australian context.

Our centre follows a targeted screening practice, with those on immunosuppressive medications or being assessed for renal transplant recommended to undergo testing for LTBI using IGRA and CXR. All patients are required to be screened prior to transplantation waitlisting (though our unit is not a transplant centre). Decision regarding screening for the remainder of the dialysis cohort is made on an individual basis by the treating physician. This audit aims to review cases of active and latent tuberculosis in patients commencing dialysis over a six-year period in order to inform unit protocol for the testing of LTBI.

## Methods

A retrospective review of new haemodialysis and peritoneal dialysis patients eighteen years and older at Western Health during the period 1 Jan 2012 to 31 Dec 2018 was undertaken. The aim was to review active TB cases and determine if current targeted screening practices for detection for LTBI in dialysis patients achieved high sensitivity of active TB cases.

### Data collection

Australian and New Zealand Dialysis and Transplant Registry Data and hospital medical records were reviewed for basic demographic data, comorbidities, CXR within 6 months of dialysis commencement, LTBI screening using Quantiferon Gold (QFG), and outcomes, including episodes of active TB to end of data collection at June 2020, allowing for 18months follow up from last patient commencing dialysis. Data was collected in Excel.

### Outcomes

The primary outcomes were cases of active TB during the study period and description of the current screening practice in a dialysis cohort. Secondary outcomes were rates of LTBI diagnosed in the screened group and factors associated with diagnosis. A sub analysis of patients from WHO high incidence country of birth (incidence of > 100 cases per 100,000 population per year) was completed to assess for potential benefits of generalised screening in this group.

### Statistical analysis

The data set was normally distributed. Categorical variables were compared using a Pearson Chi Square test. For continuous variables, a Levene’s test of equality of variances was conducted and continuous variables compared using independent T test. A p value < 0.05 was considered significant. Data was analysed using IBM SPSS (version 20.0 Chicago, IL).

## Results

472 patients commenced dialysis between 1 and 2012 and 31 Dec 2018, from 54 different birth countries with 22% (n = 103) coming from WHO high risk country of birth for TB. Patients were followed up to 30 June 2020, with a median follow up of 38 months. The average age was 61 (SD ± 14) with a male predominance 64%. 229 (48.5%) patients were screened for LTBI using QFG. (Table 1) The main indication for screening was transplantation work-up (87.8%). The predominance of screening as part of renal transplantation work up is reflected in the screened patient demographics as screened patients were more likely to be of younger age have less co-morbidities and more likely to be alive at the end of the study period (Table 1). The targeted approach to screening is also reflected in the finding that screened patients were more likely to come from a WHO high risk country of origin.

**Table 1:** Baseline characteristics, comorbidities and outcomes between groups not screened compared to screened for latent tuberculosis infection using Quantiferon Gold

		Not screened	Screened	Significance
Total		243	229	
Gender	Male	164 (67.5%)	140 (61.1%)	0.15
	Female	79 (32.5%)	89 (38.9%)	
Smoker	Never	117 (48.2%)	105 (45.9%)	0.23
	Former	97 (39.9%)	84 (36.7%)	
	Current	29 (11.9%)	40 (17.4%)	
Age		69 ± 10	52 ± 12	< 0.001
Risk in country of origin	Low < 10 per 100,000 population per year	130 (53.5%)	103 (45.0%)	> 0.05
	Intermediate 10-100 per 100,000 population per year	76 (31.3%)	56 (22.5%)	> 0.05
	High > 100 per 100,000 population per year	37 (15.2%)	70 (30.5%)	< 0.001
Lung disease		32 (13.2%)	21 (9.2%)	0.17
Coronary disease		96 (39.5%)	57 (24.9%	0.001
PVD		48 (19.8%)	33 (14.4%)	0.12
CVD		40 (16.5%)	22 (9.6%)	0.02
Diabetes		159 (65.4%)	122 (53.2%)	0.007
Immunosuppression		14 (5.8%)	23 (10.0%)	0.08
Cancer		28 (11.5%)	18 (7.9%)	0.14
Active TB	During study		2 (0.9%)	0.34
	Pre study	4 (1.7%)	4 (1.8%)	
Died		84 (34.6%)	32 (14.0%)	< 0.001

Categorical variables used Pearson Chi Square test. Continuous variables used independent T test. A Levene’s test of equality of variances was conducted

The primary outcome of active tuberculosis developed in two patients during the observation period. Both cases had prior screening using QFG in context of transplant work up. One case occurred within the first 12 months of dialysis, with a negative QGF 11 months prior to clinical diagnosis. The second case was from a high-risk country and had been screened with a negative result 16 months prior to development of active disease.

All patients were clinically reviewed prior to starting dialysis. 369 patients out of 472 had a CXR within 6 months of commencement of dialysis (76.3%). 38.4% of the results were abnormal, most commonly due to evidence of pulmonary oedema or effusions. Only 8 cases had changes reported potentially consistent with a past history of tuberculosis, with 3 cases having a known past history, 4 cases undergoing LTBI workup (for transplantation listing) and 1 case with no further investigation nor tuberculosis related outcome. Use of CXR in this cohort did not change clinical management.

There were two patients who underwent transplant work up at our service who did not have a QFG prior to transplant, noting this may have been done at the transplanting site. One patient was from a low-risk country and the other was from a high-risk country, neither developed active TB. Whilst there were no cases of post-transplant active TB in this cohort, transplant outcomes are incomplete as under two-thirds of patients were followed up at our centre.

**Fig. 1:**
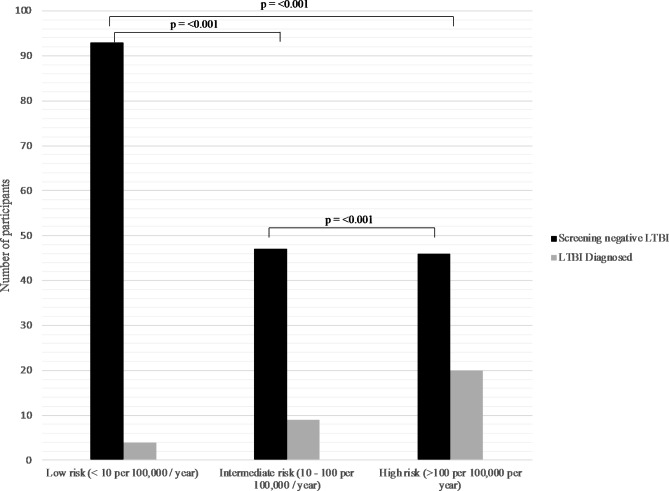
Results of Quantiferon Gold Result in participants who underwent screening, based on risk of TB in WHO defined high risk country of origin (> 100 cases per 100,000 population per year) and intermediate risk county of origin (10–100 cases per 100,000 population per year), compared to low TB risk country of origin (< 10 per 100,000 population per year). P values calculated using Chi Square demonstrate significant difference based on risk in country of origin

Table 2 summarizes the secondary outcomes comparing the population who screened negative with those diagnosed with LTBI during the study period. Importantly WHO high risk country of birth was associated with LTBI (Fig. 1). In this analysis there were 13 initial indeterminant results re-classified as negative (based on repeat testing and further work up). Another 10 indeterminant cases were either not re-screened given not proceeding to transplant, being low clinical risk or no documentation as to the result, these cases were excluded from the secondary analysis. Patients who tested positive for LTBI were reviewed by an infectious disease physician and 26 underwent latent tuberculosis treatment (in two cases this was prior to dialysis commencement). Two patients had side effects to isoniazid treatment and changed to rifampicin, and a third had an intolerance to therapy and declined an alternate regime. Of the remainder, 1 patient had already undergone tuberculosis treatment, in 4 patients a decision was made to only treat if proceeding to transplant or immunosuppression, and in two patients there was unclear documentation.

**Table 2:** Baseline characteristics, comorbidities and outcomes within the screened population comparing Quantiferon gold negative (tested negative) and Quantiferon Gold positive (tested positive) groups

		Tested negative	Tested positive	Significance
Total	219	186	33	
Gender	Male	111 (59.7%)	23 (69.7%)	0.28
	Female	75 (40.3%)	10 (30.3%)	
Smoker	Never	88 (47.3%)	13 (39.4%)	0.08
	Former	61 (32.8%)	17 (51.5%)	
	Current	37 (19.9%)	3 (9.1%)	
Age		51 ± 12.7	56 ± 10.9	0.03
Risk in country of origin	Low < 10 per 100,000 population per year	93 (50.0%)	4 (12.0%)	< 0.001
	Intermediate 10-100 per 100,000 population per year	47 (25.3%)	9 (27.3%)	
	High > 100 per 100,000 population per year	46 (24.7%)	20 (60.6%)	
Lung disease		17 (9.1%)	0 (0.0%)	0.07
Coronary disease		43 (23.1%)	11 (33.3%)	0.21
PVD		25 (13.4%)	7 (21.2%)	0.24
CVD		18 (9.7%)	4 (12.1%)	0.66
Diabetes		102 (54.8%)	16 (48.5%)	0.50
IS		18 (9.7%)	1 (3.0%)	0.21
Cancer		16 (8.6%)	1 (3.0%)	0.27
Active TB		2 (1.1%)	0 (0.0%)	0.73
Died		26 (13.9%)	2 (6.1%)	0.20

Categorical variables used Pearson Chi Square test. Continuous variables used independent T test. A Levene’s test of equality of variances was conducted

WHO high risk country of origin was associated with being diagnosed with latent Tuberculosis with an odds ratio 10.4 (95% CI 3.3–31.2) compared to coming from a low-risk birth country.

Further sub-analysis was conducted on the patients who had high risk country of birth (Table 3). Those not tested were significantly older in age and more likely to die over the follow up period. There were no adverse tuberculosis outcomes in those not tested from high-risk country of origin, with median follow up 50 months in those who survived to end of follow-up.

**Table 3:** Baseline characteristics, comorbidities and outcomes in patients from high risk country of origin for TB (> 100 cases per 100,000 population per year) comparing those not screened with screened for LTBI using QFG

		Not screened	Screened	Significance
Total		37	70	
Gender	Male	17 (45.9%)	45 (64.3%)	0.07
	Female	20 (53.1%)	25 (35.7%)	
Smoker	Never	26 (70.3%)	37 (52.5%)	0.31
	Former	8 (21.6%)	25 (35.7%)	
	Current	2 (5.4%)	8 911.4%)	
Age		71 ± 8	52 ± 11	< 0.01
Lung disease		3 (8.1%)	5 (7.1%)	0.86
Coronary disease		10 (27%)	15 (21.4%)	0.5
PVD		4 (10.8%)	10 (14.3)	0.61
CVD		4 (10.8%)	4 (5.7%)	0.34
Diabetes		25 (67.6%)	38 (54.3%)	0.18
IS		1 (2.7%)	5 (7.2%)	0.33
Cancer Diagnosis		3 (8.1%)	2 (2.9%)	0.22
Active TB	During study	0	1 (1.4%)	0.56
	Pre study	3 (8.1%)	3 (4.2%)	
Death		10 (27%)	6 (8.7%)	0.01

Categorical variables used Pearson Chi Square test. Continuous variables used independent T test. A Levene’s test of equality of variances was conducted

## Discussion

Tuberculosis is a devastating disease which contributes to morbidity and mortality throughout the world. The WHO End TB strategy systematic testing and treatment for LTBI in at risk cohorts including dialysis patients for high- and middle-income countries is recommended [7]. The aim is to identify subgroups who have greater risk of developing active TB, such that testing and treatment provides greater benefit than harm [7]. Importantly, the guidelines acknowledge this recommendation is based on very low-quality evidence [7]. In contrast several other guidelines follow a more targeted approach [8–15] and suggest testing on an intent to treat basis [9, 11–15].

The findings of this retrospective cohort study support a targeted approach, with no cases of active TB in the non-screened cohort. It was the objective of this study to examine QFG based screening. Therefore, this study cannot make recommendations regarding screening with use of the TST. Both cases of active TB occurred in the cohort who screened negative on QFG, either indicating a false negative QFG result, or exposure and development of active TB post dialysis commencement. This reflects a low false negative rate of QFG and is consistent with previous research [16].

The main limitation of our study was that it was a retrospective study with a small cohort. Even in TB endemic countries the incidence of this condition is still only > 100/100,000 person per year and thus a much larger sample size would be required to demonstrate a benefit to a generalised screening approach. A second limitation is that this was a retrospective record review, and some patients may have had screening externally that is not captured in the data.

Another finding was that in our cohort the use of CXR, as suggested by the British Thoracic Guidelines [8], did not meaningfully change LTBI screening decisions. Potential reasons for this include the predominance of fluid overload on CXR (38.4%) and secondly it was unknown if the question of LTBI was raised in the requesting information.

Decision to screen aligned strongly with physician assessment of potential transplant eligibility, with 87.8% being screened for transplant assessment. This is reflected in a younger age in those screened, however older patients were more likely to screen positive. Importantly, the strongest association with a diagnosis of LTBI was WHO high risk country of origin. This raises the question of whether this group should have routine QFG screening prior to commencement of dialysis. In our sub-analysis of the 37 non-screened patients from high-risk country of origin, the average age was 71 years and over a quarter (27%) died during the study period from non-TB related causes. The remainder of this subset had a median follow up time of 50 months without development of active TB. Based on the older age, absence of active TB development and the poor life expectancy of the older dialysis cohort we predict that increasing screening to this subgroup was unlikely to have resulted in benefit and potentially contributed to harm. Importantly screening for LTBI comes with the risk of false positive results and treatment requires further investigations, multiple clinical appointments, and monitoring, in addition to potential toxicities and drug interactions associated with the prolonged treatment course [3, 8].

A cohort study conducted in British Columbia where routine screening is now standardised demonstrated a small reduction in overall cases of active TB using a generalised approach [17]. However, further study of this cohort reviewed outcomes for LTBI treatment and found that found over 20% of those treated for LTBI had a grade 3 or 4 adverse effect [18]. Their analysis also concluded that the success of their program and prevention of more serious adverse effects was due to close follow up including monthly appointments, an important consideration in any change in screening protocols [18]. Of note, we had two patients need to change therapy due to adverse side effects, and a third cease secondary to intolerance. However, in comparison we had a much smaller sample size, and our screening had a propensity for younger patients with less comorbidities who are more likely to tolerate treatment.

In summary the risk of TB in dialysis patient has been consistently shown to be increased, though the absolute risk in those with no other risk factors is lower, particularly in those from non-endemic areas, and therefore the justification and potential harms in testing all Australian dialysis patients need to be assessed in considering adoption of the WHO guidelines. Screening for LTBI in patients born in high-risk country of origin would offer greater yield, but needs to take into consideration intent to offer preventative treatment. As our study demonstrates the decision to offer treatment will be dictated by individual factors including co-morbidities, frailty and life expectancy.

Routine testing of all dialysis patients may or may not prove beneficial for the individual patient or the wider Australian community. In order to better inform Health policy with regards to screening for LTBI in the Australian dialysis cohort further research analysing all Australian dialysis unit screening and treatment practices for LTBI and the impact on patient outcomes would be required. Specifically, evaluation of TB related outcomes and implications of screening, including treatment side effects and costs, would help guide practice.

## Conclusion

Retrospective analysis of latent Tuberculosis screening in an Australian tertiary dialysis centre with patients of a diverse ethnic background, failed to show a potential benefit from widening of the current screening program within our unit, however a much larger sample size would be required to show a potential screening benefit. The data did confirm that WHO high risk country of origin was associated with latent TB diagnosis. Further analysis of Australian dialysis units is warranted to ascertain the benefits if any of widening the screening program across Australia to include all dialysis patients as recommended by the WHO.

## Data Availability

The authors declare that the data supporting the findings of this study are available within the article. Any further data is available from HW but is not publicly available due to participant privacy.

## List of abbreviations

TB: Tuberculosis
LTBI: Latent Tuberculosis infection
WHO: World Health Organisation
QFG: Quantiferon Gold
TST: Tuberculin skin test
IGRA: Interferon gamma release assay
CXR: Chest X-ray
